# OTX2 exhibits cell-context-dependent effects on cellular and molecular properties of human embryonic neural precursors and medulloblastoma cells

**DOI:** 10.1242/dmm.020594

**Published:** 2015-10-01

**Authors:** Ravinder Kaur, Christopher Aiken, Ludivine Coudière Morrison, Radhika Rao, Marc R. Del Bigio, Shravanti Rampalli, Tamra Werbowetski-Ogilvie

**Affiliations:** 1 Regenerative Medicine Program, Departments of Biochemistry & Medical Genetics and Physiology & Pathophysiology, University of Manitoba, Winnipeg, Manitoba, Canada, R3E 0J9; 2 Centre for Inflammation and Tissue Homeostasis, Institute for Stem Cell Biology and Regenerative Medicine (inStem), NCBS-TIFR Campus, GKVK PO, Bellary Road, Bangalore 560065, India; 3 Department of Pathology, University of Manitoba, 401 Brodie Centre, 727 McDermot Avenue, Winnipeg, Manitoba, Canada, R3E 3P5; 4 Children's Hospital Research Institute of Manitoba, Winnipeg, Manitoba, Canada

**Keywords:** Human embryonic stem cells, hESCs, OTX2, Brain tumor modeling, Medulloblastoma

## Abstract

Medulloblastoma (MB) is the most common malignant primary pediatric brain tumor and is currently divided into four subtypes based on different genomic alterations, gene expression profiles and response to treatment: WNT, Sonic Hedgehog (SHH), Group 3 and Group 4. This extensive heterogeneity has made it difficult to assess the functional relevance of genes to malignant progression. For example, expression of the transcription factor Orthodenticle homeobox2 (OTX2) is frequently dysregulated in multiple MB variants; however, its role may be subtype specific. We recently demonstrated that neural precursors derived from transformed human embryonic stem cells (trans-hENs), but not their normal counterparts (hENs), resemble Groups 3 and 4 MB *in vitro* and *in vivo*. Here, we tested the utility of this model system as a means of dissecting the role of OTX2 in MB using gain- and loss-of-function studies in hENs and trans-hENs, respectively. Parallel experiments with MB cells revealed that OTX2 exerts inhibitory effects on hEN and SHH MB cells by regulating growth, self-renewal and migration *in vitro* and tumor growth *in vivo*. This was accompanied by decreased expression of pluripotent genes, such as *SOX2*, and was supported by overexpression of SOX2 in OTX2+ SHH MB and hENs that resulted in significant rescue of self-renewal and cell migration. By contrast, OTX2 is oncogenic and promotes self-renewal of trans-hENs and Groups 3 and 4 MB independent of pluripotent gene expression. Our results demonstrate a novel role for OTX2 in self-renewal and migration of hENs and MB cells and reveal a cell-context-dependent link between OTX2 and pluripotent genes. Our study underscores the value of human embryonic stem cell derivatives as alternatives to cell lines and heterogeneous patient samples for investigating the contribution of key developmental regulators to MB progression.

## Abstract (precis)

**Summary:** Human embryonic stem cell neural derivatives can be used to model the molecular and cellular properties of medulloblastoma.

## INTRODUCTION

Embryonal tumors of the central nervous system constitute a group of highly malignant cancers that manifest early during development and include medulloblastoma (MB), primitive neuro-ectodermal tumors and atypical rhabdoid/teratoid tumor (Mehta et al., 2011). Following surgery and treatment with chemotherapy or radiation, these tumors frequently recur as a consequence of tumor cell infiltration into the brain and/or metastasis through the cerebral spinal fluid. Despite numerous advances in our understanding of the molecular basis for the malignant state and improved 5-year survival rates, particularly for MB (Mehta et al., 2011), children typically suffer from permanent cognitive and physical dysfunction attributed to the long-term toxicities of therapies on their developing nervous systems.

Recent advances in genomic sequencing and microarray technologies have revolutionized our understanding of pediatric brain tumor heterogeneity. For example, MB consists of four distinct subtypes exhibiting different genomic alterations, gene expression profiles and response to treatment: WNT, Sonic Hedgehog (SHH), Group 3 and Group 4 (Taylor et al., 2012). This had led to the identification of many subgroup-specific mutated genes (Jones et al., 2012; Northcott et al., 2012; Pugh et al., 2012; Robinson et al., 2012); however, the functional role of both mutated and differentially expressed genes is poorly understood. Following completion of the ‘omics’ analyses, future studies must determine the role of these genes in the maintenance and progression of MB subtypes. This will be particularly difficult, as the functional relevance of candidate genes will not be universal for all MB variants and may have to be considered in a subtype-specific manner. Indeed, studying the role of these genes in well-established murine models will provide greater insight, and even *Drosophila melanogaster* has recently been used to identify novel genes associated with pediatric brain tumors, such as atypical rhabdoid/teratoid tumor (Jeibmann et al., 2014). Nevertheless, complementary human models are still needed to both verify and identify the functional relevance of specific genes to pediatric neural tumor progression.

We previously compared an established normal human embryonic stem cell (hESC) cell line (H9; Thomson et al., 1998) with multiple ‘transformed’ subclones derived from the same cell line (trans-hESCs) that had spontaneously acquired features of neoplastic progression (Werbowetski-Ogilvie et al., 2009). Normal pluripotent hESC lines are routinely evaluated for transformation and acquisition of neoplastic properties based on a variety of well-defined parameters including, but not limited to, growth factor independence, decreased differentiation and adoption of abnormal karyotypes (Werbowetski-Ogilvie et al., 2009). Follow-up studies with neural precursors derived from trans-hESCs, herein called trans-hENs, demonstrated that these cells resemble human Group 3 and 4 MB *in vivo* (Werbowetski-Ogilvie et al., 2012). Global gene expression analysis revealed differential expression of 1346 transcripts in trans-hENs versus hENs, including upregulation of both a pluripotency and an MB transcription program that exhibited similarities to Groups 3 and 4.

**TRANSLATIONAL IMPACT**

**Clinical issue**

Recent advances in genomic sequencing and microarray technologies have heightened our understanding of the extensive molecular and genetic heterogeneity that underlie highly aggressive pediatric brain tumors. For example, medulloblastoma (MB) consists of four distinct subtypes – called WNT, Sonic Hedgehog (SHH), Group 3 and Group 4 – which exhibit different genomic alterations, gene expression profiles and response to treatment. This has led to the identification of many subgroup-specific genes that are mutated or differentially expressed in these MB subgroups; however, the role of these genes in the progression of MB subtypes is still unexplored. To investigate this, the functional relevance of candidate genes has to be considered in a subtype-specific manner, taking MB heterogeneity into account. In this paper, the authors use neural derivatives from human embryonic stem cells (hESCs) as a model for studying the role of the homeodomain transcription factor orthodenticle homeobox 2 (OTX2) in the MB subgroups both *in vitro* and *in vivo*. OTX2 is of particular interest as previous studies on the role of OTX2 in MB cell lines have demonstrated conflicting results, showing differential effects on cell growth, thus suggesting that the function of OTX2 might depend on the cell context and on the MB subtype.

**Results**

By employing hESC derivatives as a powerful cell resource for investigating key biological questions related to MB, the authors sought to evaluate the role of OTX2 in MB cells using gain- and loss-of-function approaches both *in vitro* and *in vivo*. In particular, it was found that OTX2 exerts inhibitory effects on human embryonic neural precursors (hENs) and in SHH MB cells by decreasing self-renewal, migration and growth, probably through regulation of pluripotent genes, such as SOX2. By contrast, OTX2 was oncogenic or promoted self-renewal of trans-hENs and Groups 3 and 4 MB, but this effect was independent of pluripotent genes.

**Implications and future directions**

These results revealed novel cell-context-dependent functions of OTX2 and underscored the value of hESC derivatives as an alternative to cell lines and heterogeneous patient samples for investigating the contribution of key developmental regulators of MB progression. In addition, neural derivatives from hESCs represent a biologically relevant human model system enabling comprehensive studies of the balance between self-renewal, proliferation and differentiation. Defining the molecules and pathways that regulate these processes will have important implications not only for neurodevelopment but also for understanding the fundamental processes contributing to pediatric brain tumor initiation and progression.

One of the transcripts upregulated in trans-hENs (>12-fold) is orthodenticle homeobox 2 (*OTX2*; Werbowetski-Ogilvie et al., 2012). As a member of the highly conserved bicoid homeodomain transcription factor family that also includes OTX1 and CRX, OTX2 has been shown to play crucial roles in brain, cerebellum, pineal gland and eye development (Acampora et al., 1995; Matsuo et al., 1995; Ang et al., 1996; Simeone, 1998; Beby and Lamonerie, 2013). Notably, complete knockout of mouse *Otx2* is embryonic lethal and results in the deletion of both forebrain and midbrain regions. This is known as the ‘headless phenotype’ and is attributed to defective anterior neuroectoderm specification during gastrulation (Acampora et al., 1995). Heterozygous mice have been shown to exhibit craniofacial malformations, such as anophthalmia/microphthalmia (absent or small eyes), short nose or agnathia/micrognathia (absent or small jaw; Matsuo et al., 1995). Otx2 has also been shown to play a pivotal role in defining the boundary between midbrain and hindbrain as the isthmic organizer (Broccoli et al., 1999). Ectopic expression of *Otx2* across the midbrain-hindbrain barrier into the anterior hindbrain results in deletion of anterior cerebellar regions and expansion of posterior midbrain (Broccoli et al., 1999), demonstrating that Otx2 is essential for patterning and formation of the rostral brain. During the later stages of human cerebellar development, OTX2 is expressed in the progenitor cells of the external granular layer but is not detected at the postnatal stage (de Haas et al., 2006). In the postnatal cerebellum, OTX2 levels become negligible as expression is restricted to choroid plexus, pineal gland and retinal pigment epithelium in adult tissues (Fossat et al., 2006). Primary MBs most often develop in the cerebellum, and OTX2 is amplified and overexpressed in more than 60% of cases (Michiels et al., 1999; Boon et al., 2005; Di et al., 2005; de Haas et al., 2006). Higher levels are seen particularly in Groups 3 and 4, whereas its expression is negligible in the SHH variant (Bunt et al., 2010). Studies evaluating the function of OTX2 in MB have demonstrated conflicting results. For example, OTX2 has been shown to play an oncogenic role in maintaining cell growth of Group 3 and 4 MB cell lines (Di et al., 2005; Adamson et al., 2010). However, one study evaluating OTX2 overexpression in SHH MB lines revealed that OTX2 suppresses cell proliferation and induces cell senescence specifically *in vitro* (Bunt et al., 2010). Even in the nervous system, OTX2 maintains ventral mesencephalon progenitor cell proliferation (Omodei et al., 2008), whereas it appears to inhibit proliferation in the thalamus (Puelles et al., 2006). These opposing data suggest that the effect of OTX2 on cell growth may be dependent on the cell type and neuroanatomical region. This concept has not yet been tested directly in MB cells. Furthermore, previous studies focused on cell proliferation/survival of MB cells and did not evaluate other cellular properties, including cell migration and aspects of stem cell function such as self-renewal. This is important because invasive growth and metastasis contribute to recurrence and poor prognosis in MB patients (Mehta et al., 2011). Moreover, brain tumor stem cell populations have been shown to be critical for MB propagation and maintenance (Singh et al., 2003, 2004). Here, we tested the utility of trans-hENs and hENs as potential surrogates for heterogeneous MB cells by evaluating the role of OTX2 using gain- and loss-of-function studies *in vitro* and *in vivo*. Using this model system, we demonstrated that OTX2 plays a novel role in self-renewal and migration and a context-dependent regulation of pluripotency genes, such as *SOX2*. Our study supports the use of neural derivatives from hESCs as a model system for studying the cellular and molecular mechanisms that contribute to MB progression.

## RESULTS

### Overexpression of OTX2 decreases cell growth in hENs and Daoy medulloblastoma cells

**Fig. 1.**
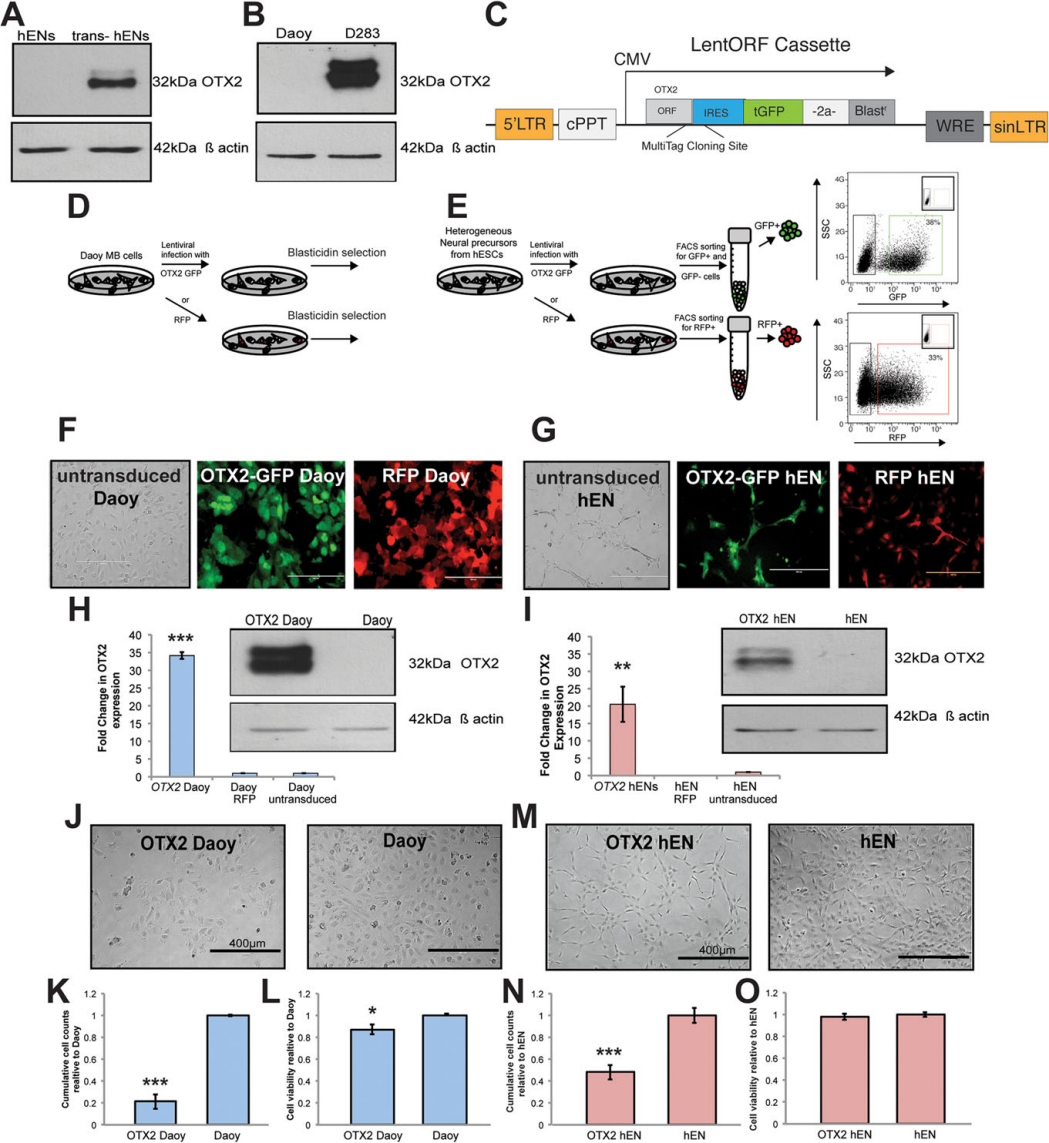
**Overexpression of OTX2 in Daoy and hEN cells decreases cell growth.** (A,B) Endogenous protein levels of OTX2 in hEN versus trans-hEN cultured in neural precursor conditions for 7 days (A) and Daoy (SHH MB variant) versus D283 (Group 4 MB variant; B). β-actin serves as a loading control. (C) LentiORF cassette used for stable overexpression of OTX2 in hEN and Daoy cells. (D,E) Schematic representation of stable Blasticidin selection for OTX2-Daoy and RFP-Daoy cells (D) and sorting procedure used to distinguish between infected (GFP+) and non-infected (GFP−) hEN cells as well as RFP+ control hENs (E). (F,G) Blasticidin-selected Daoy populations (F) and sorted hEN cell populations (G) used for cell proliferation, self-renewal and migration assays *in vitro*. Scale bars: 200 µm. (H,I) Validation of stable OTX2 overexpression in Daoy (H) and hEN (I) by qPCR and western blot. β-actin serves as a loading control. Error bars: s.e.m. **P<0.01, ***P<0.001. (J-O) Overexpression of OTX2 reduces cell number in both Daoy (J-L) and hEN cells (M-O). In both cell lines, OTX2 overexpression significantly decreases total cell number (J,K,M,N), with only a small change in cell viability (L,O). Scale bars: 400 µm. Error bars: s.e.m. *P<0.05, ***P<0.001. For all experiments, *n*=3 biological replicates or independent infections.

Our previous studies revealed increased OTX2 transcript levels in trans-hENs relative to hENs (Werbowetski-Ogilvie et al., 2012). In addition, OTX2 expression is increased in Group 3 and 4 MB cell lines (i.e. D341 and D283, respectively) and is negligible or absent in representative SHH MB variant lines, such as Daoy (Bunt et al., 2010). To confirm differential expression between these sets of cell lines, we evaluated OTX2 levels by western blot (Fig. 1A,B). OTX2 was not detected in hENs that had been cultured in neural precursor media for 7 days; however, levels were still high in trans-hENs (Fig. 1A). Likewise, OTX2 was not detected in Daoy cells; however, protein levels were high in D283 (Fig. 1B). These paired cell lines were therefore used for all parallel gain- and loss-of-function studies. For stable OTX2 overexpression, we employed LentiORFs consisting of dual-expression constructs with TurboGFP as a marker to track transduction efficiency in hENs and Daoy (Fig. 1C). LentiORF RFP-expressing viral particles were used as a control for each cell line. Following selection of GFP+ and RFP+ cells (Fig. 1D-G), OTX2 overexpression was verified by both qPCR and western blot in OTX2+ Daoy and hEN (Fig. 1H,I). For both cell lines, OTX2 expression was negligible for RFP+ and untransduced cells (Fig. 1H,I); therefore, we refer hereafter to Daoy-RFP+ and hEN-RFP+ as Daoy and hEN, respectively.

We first compared cumulative cell growth and viability in OTX2+ cells versus controls (Fig. 1J-O). Compared with Daoy cells, OTX2+ Daoy exhibit a significant decrease in cell number over 4 days (Fig. 1J,K). This was not entirely attributed to cell death, as OTX2+ cells displayed only a 20% decrease in total live cells by Trypan Blue staining and no significant change by Annexin V staining (Fig. 1L; supplementary material Fig. S1A,B). Similar results were obtained for hENs, as overexpression of OTX2 resulted in a 60% decline in total cell number without significantly affecting cell viability (Fig. 1M-O, supplementary material Fig. S1C,D). Bromodeoxyuridine (BrdU) staining supported our findings and demonstrated a decrease of cells in S phase and an increase in G0/G1 following OTX2 overexpression (supplementary material Fig. S1E). We did not observe a change in the frequency of apoptotic cells (supplementary material Fig. S1E). These results confirm previous findings obtained for Daoy MB cells (Bunt et al., 2010), but, more importantly, demonstrate that overexpression of OTX2 in hENs also recapitulates the inhibitory effect on cell growth.

### Overexpression of OTX2 in Daoy and hENs decreases self-renewal, survival and cell migration

**Fig. 2.**
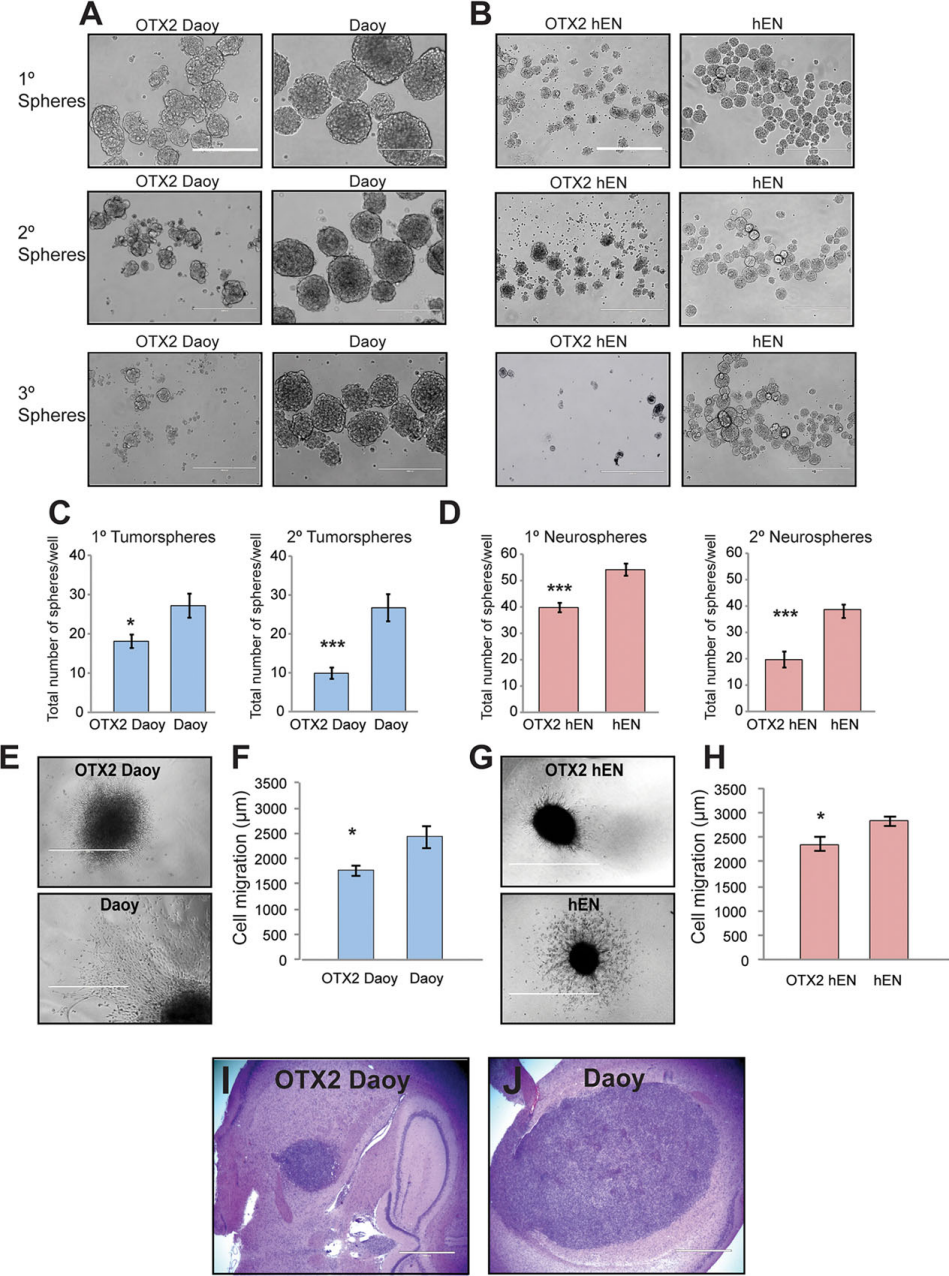
**Overexpression of OTX2 significantly decreases self-renewal and migration *in vitro* and tumor growth *in vivo.*** (A,B) Representative images of spheres over subsequent passage following OTX2 overexpression in Daoy (A) and hEN cells (B). (C,D) OTX2 overexpression decreases self-renewal capacity in both Daoy (C) and hEN spheres (D). Error bars: s.e.m. *P<0.05, ***P<0.001. Scale bars: 400 µm. (E-H) OTX2 overexpression decreases migration in collagen gels for both Daoy (E,F) and hENs (G,H). Error bars: s.e.m. *P<0.05. (E,G) Representative images of Daoy (E) and hEN aggregates (G) in collagen over 3 days following OTX2 overexpression. Scale bars: 1000 µm. For all experiments, *n*=3 biological replicates or independent infections. (I,J) Tumor growth of OTX2+ Daoy relative to Daoy cells in NOD SCID mice (*n*=5 for OTX2+ Daoy and *n*=3 for Daoy). Scale bars: 1000 µm.

We next evaluated the effect of OTX2 overexpression on self-renewal capacity and migration (Fig. 2A-H). OTX2+ and control cells were subjected to neurospheres/tumorsphere assays and the number of spheres assessed over subsequent passage (Fig. 2A-D). Compared with Daoy, OTX2+ Daoy displayed a significantly decreased capacity for self-renewal (Fig. 2A,C). However, we also observed a significant drop in cell viability with each passage by Trypan Blue and Annexin V staining (supplementary material Fig. S2A,C-F). This was supported by BrdU staining that demonstrated a concomitant decrease in S phase and increase in apoptotic cells following OTX2 overexpression (supplementary material Fig. S3). Similar results were obtained for hENs, as OTX2+ hENs displayed a lower capacity for self-renewal (Fig. 2B,D) and a significant reduction in cell viability (supplementary material Fig. S2B,G-J). These results demonstrate that OTX2 plays a novel role in regulating stem cell properties of both SHH MB cells and hENs and that cell survival contributes to these adverse effects in stem cell-enriched conditions.

To evaluate cell migration, we prepared hanging drops from OTX2+ and control cells from both cell lines and evaluated cell motility in collagen gels. Compared with respective controls, both OTX2+ Daoy and OTX2+ hENs exhibit a significant decline in cell migration (Fig. 2E-H) over 72 h. Taken together, these results indicate that OTX2 overexpression results in a general repressive effect on all cellular properties examined *in vitro* for both SHH MB cells and hENs.

### OTX2 overexpression decreases tumor growth *in vivo*

Although Bunt et al. (2010) previously evaluated the effect of OTX2 overexpression on SHH MB cell line growth *in vitro*, they did not determine the effect on tumor growth *in vivo*. To compare the tumorigenic potential of OTX2+ Daoy and Daoy *in vivo*, 2.5×10^5^ cells for each line were injected into the right frontal lobe of non-obese diabetic severe combined immunodeficient (NOD SCID) mice and examined for tumor formation. As hENs are normal cells not amenable to tumor formation (Werbowetski-Ogilvie et al., 2012) and all cellular properties were reduced following OTX2 overexpression, we did not inject hENs and OTX2+ hENs into NOD SCID mice. Compared with Daoy, mice injected with OTX2+ Daoy cells displayed smaller tumors (Fig. 2I,J) but were histologically similar to controls. Tumors were well circumscribed and consisted of large pleomorphic cells, with small nodules also visible along the lateral and third ventricles (Fig. 2I,J). These results support our *in vitro* findings and demonstrate that OTX2 overexpression also suppresses Daoy MB tumor growth *in vivo*.

### Global gene expression analysis reveals downregulation of transcripts associated with cell proliferation, migration and pluripotency following OTX2 overexpression

**Fig. 3.**
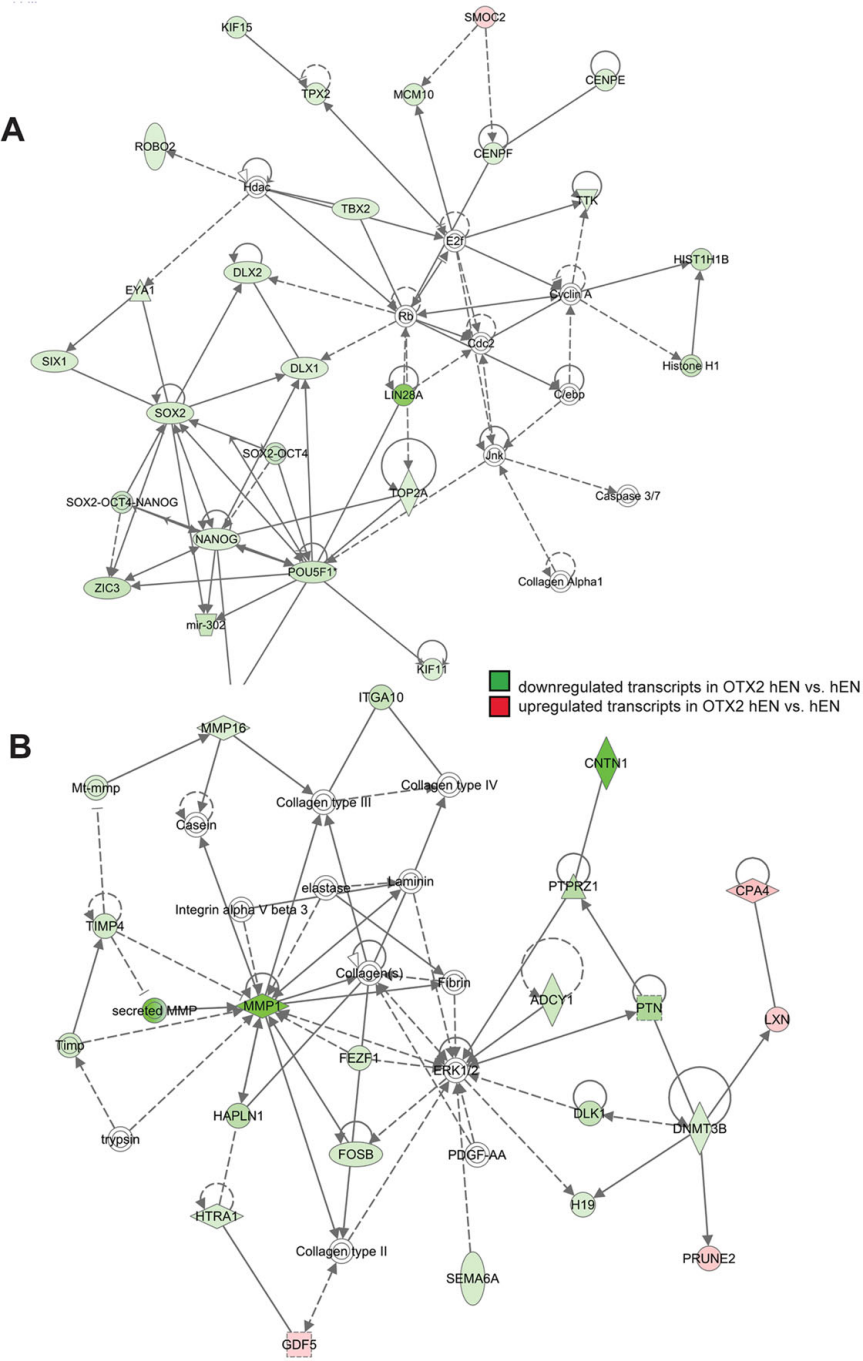
**OTX2 overexpression results in dysregulation of transcriptome networks associated with cell proliferation and cell motility.** Top dysregulated cellular networks consisting of transcripts associated with cell proliferation (A) and cell migration (B). Shaded green areas denote transcripts that are significantly downregulated and red areas denote significantly upregulated transcripts in OTX2 hENs versus hENs. Note that the vast majority of transcripts are downregulated in OTX2 hENs. *n*=3 biological replicates or independent infections.

**Fig. 4.**
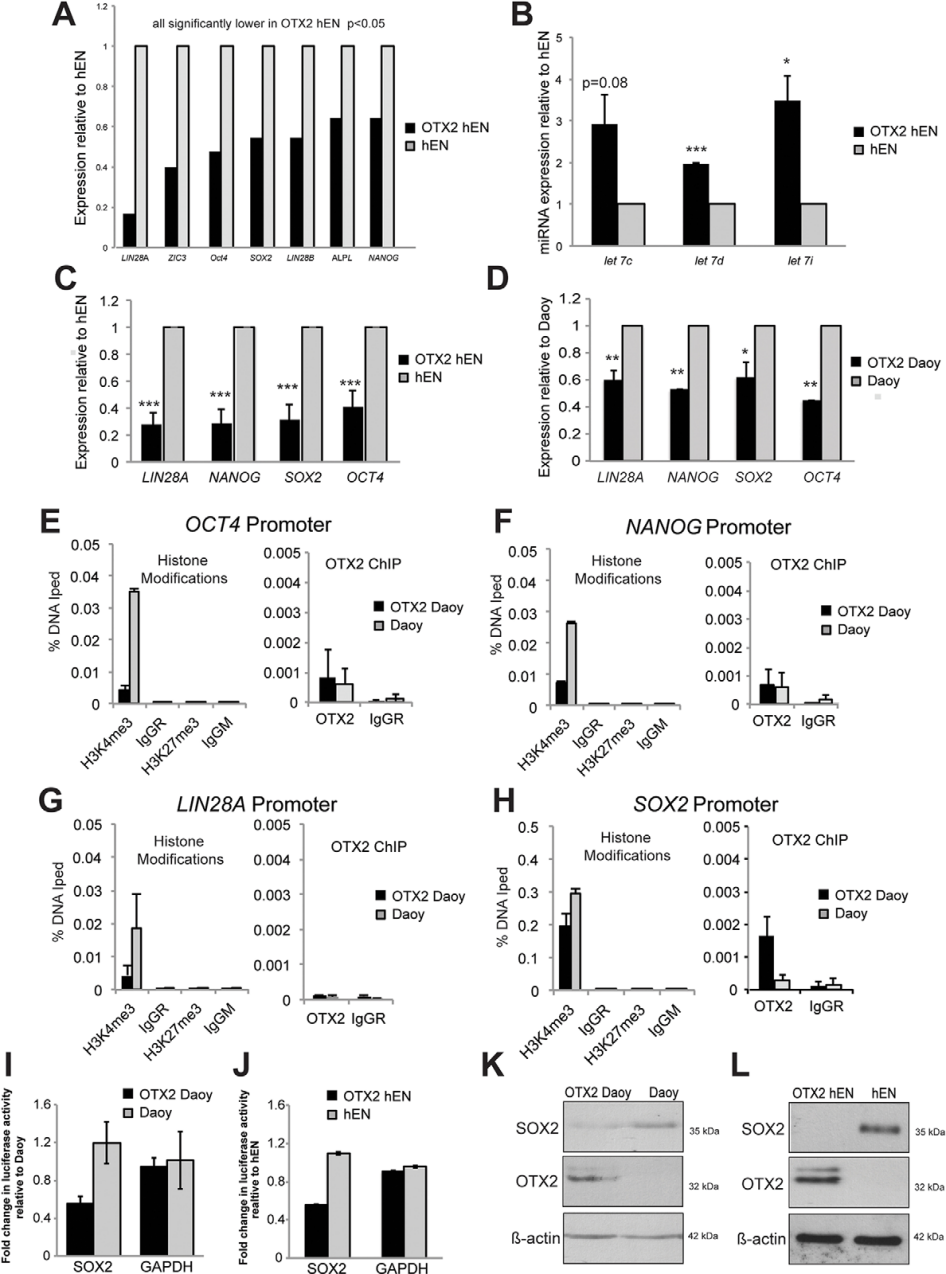
**OTX2 overexpression significantly decreases levels of hESC genes.** (A) Results from global gene expression analysis demonstrating overall downregulation of hESC pluripotency genes in OTX2-overexpressing hENs. Graph represents *n*=3 biological replicate pairs of OTX2 hEN and hENs. All transcripts are significantly different. (B) Upregulation of representative miRNA targets of LIN28A following OTX2 overexpression in hENs. *n*=3 independent biological replicates. (C,D) Validation of hESC transcript downregulation in hENs (C) and Daoy (D) following OTX2 overexpression by qPCR. Error bars: s.e.m. *P<0.05, **P<0.01, ***P<0.001. (E-H) Comparative ChIP-qPCR analysis for OTX2 binding and histone modifications (H3K4me3 and H3K27me3) on the *OCT4*, *NANOG*, *LIN28A* and *SOX2* promoters in OTX2+ Daoy versus Daoy cells. Error bars: s.d. (I,J) Luciferase activity in OTX2+ Daoy (I) and OTX2+ hEN (J) following transfection of a SOX2 promoter reporter construct. Error bars: s.e.m. Luciferase activity was normalized relative to control Daoy or hEN. (K,L) Western blots depicting decreased levels of SOX2 in Daoy SHH MB (K) and hEN cells (L) following OTX2 overexpression. β-actin serves as a loading control.

We next wanted to investigate the molecular mechanisms that contribute to the overall suppressive effect of OTX2 on cell properties in both Daoy and hEN cells. We conducted global gene expression analysis comparing the molecular profiles specifically in OTX2+ hENs relative to hENs. Affymetrix analysis revealed that a total of 319 transcripts were significantly and differentially expressed in OTX2+ hENs versus hENs (supplementary material Table S1). Of these 319 transcripts, 238 (75%) were downregulated (supplementary material Table S1). Interacting transcripts associated with cell proliferation and motility represented the top dysregulated networks (Fig. 3A,B). Based on the known function and directional changes of the differentially expressed cell proliferation and cell motility transcripts, these properties were predicted to be decreased in OTX2+ hENs (supplementary material Tables S2 and S3), providing further support for our cell function studies. From the cell proliferation and motility transcript lists, we validated decreased expression of *FOS*, *FOSB*, *IGF1*, *MMP1*, *TIMP4*, *CNTN1* and *UNC5C* by qPCR in OTX2+ hEN and OTX2+ Daoy cells (supplementary material Fig. S4). However, we did not observe a difference in *NRG1* expression between OTX2+ hEN and OTX2+ Daoy cells relative to their respective controls (data not shown). Interestingly, within the top dysregulated networks, transcripts associated with hESC function and pluripotency, including *SOX2*, *OCT4*, *NANOG* and *LIN28A*, were also significantly downregulated in OTX2+ hENs (Figs 3A and 4A). This was accompanied by concomitant significant increases in major *LIN28A* target miRNAs such as *let-7i*, *let-7d* and *let-7c* (Fig. 4B). Downregulation of hESC transcripts was confirmed by qPCR for both OTX2+ hEN and OTX2+ Daoy relative to controls (Fig. 4C,D). To test whether OTX2 associates with specific DNA binding sites present within the promoter region of candidate hESC genes (*OCT4*, *SOX2*, *NANOG* and *LIN28A*), we performed chromatin immunoprecipitation (ChIP) assays specifically in OTX2+ Daoy and Daoy cells, because it has been difficult to collect sufficiently high OTX2+ hEN cell numbers required to perform the procedure. OTX2 does not localize to the promoter regions of *OCT4*, *NANOG* and *LIN28A* (Fig. 4E-G); however, we observed an increase in OTX2 recruitment on the *SOX2* loci in OTX2+ Daoy relative to Daoy (Fig. 4H). To evaluate the functional significance of this interaction, luciferase reporter gene assays were performed. Co-transfection of OTX2+ Daoy and OTX2+ hEN cells with a SOX2 promoter reporter construct resulted in decreased luciferase expression relative to Daoy and hEN controls (Fig. 4I,J).

In support of our expression profile studies and OTX2 ChIP data, H3K4me3 activating histone modifications were strongly downregulated on *OCT4*, *NANOG* and *LIN28A* promoter loci in OTX2+ Daoy compared with Daoy cells (Fig. 4E-G). We observed a 30% reduction in H3K4me3 marks on the *SOX2* promoter in OTX2-overexpressing cells (Fig. 4H). We did not see any changes in H3K27 repressive histone modifications. We also observed a decrease in SOX2 protein levels following OTX2 overexpression in Daoy and hENs (Fig. 4K,L). Collectively, these data support our cellular studies and reveal novel functional interactions between OTX2 and SOX2 in human cells.

### SOX2 overexpression in OTX2+ Daoy and hENs rescues self-renewal and migration deficits

**Fig. 5.**
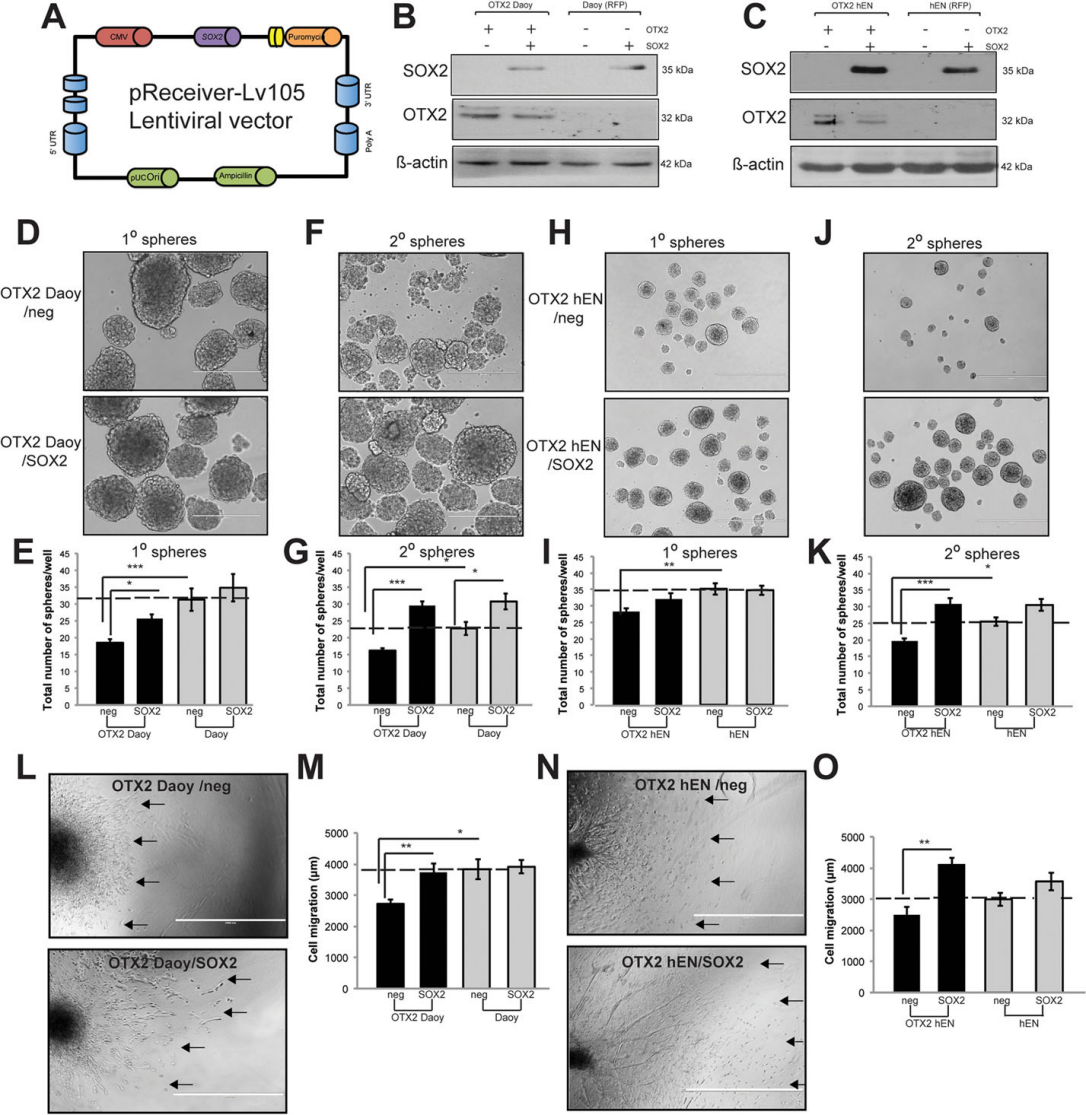
**Overexpression of SOX2 in OTX2+ Daoy and hEN cells rescues self-renewal and migration deficits.** (A) pReceiver-Lv-105 vector construct used for stable overexpession of SOX2 in hEN and Daoy SHH MB cells in the presence or absence of OTX2 overexpression. (B,C) Western blots depicting overexpression of SOX2 in Daoy SHH MB (B) and hEN cells (C) in the presence or absence of OTX2 overexpression. (D-K) SOX2 overexpression in OTX2+ cells results in a significant recovery in self-renewal for both Daoy (D-G) and hEN (H-K). (D,F) Representative images of spheres over subsequent passage following SOX2 overexpression in OTX2+ Daoy. (H,J) Representative images of spheres over subsequent passage following SOX2 overexpression in OTX2+ hENs. Scale bars: 400 µm. (L-O) SOX2 overexpression in OTX2+ cells rescues cell migration deficits in both Daoy (L,M) and hEN (N,O). Error bars: s.e.m. *P<0.05, **P<0.01, ***P<0.001. (L,N) Representative images of OTX2+ Daoy (L) and OTX2+ hEN aggregates (N) in collagen over 3 days following SOX2 overexpression. Arrows denote migration front for representative aggregates. *n*=3 biological replicates. Scale bars: 1000 µm.

SOX2+ tumor propagating cells have recently been shown to drive cell growth specifically in SHH MB mouse tumors (Ahlfeld et al., 2013; Vanner et al., 2014). Given the downregulation of SOX2 following OTX2 overexpression in our model systems, we wanted to determine whether introduction of exogenous SOX2 could rescue the cell deficits in OTX2+ SHH MB and hENs. Therefore, we stably overexpressed SOX2 in OTX2+ Daoy, OTX2+ hEN and their respective controls (that subsequently lose SOX2 expression following extended culture; Fig. 5A-C). Interestingly, following SOX2 overexpression, we observed a decrease in OTX2 levels for both OTX2+ Daoy and OTX2+ hEN, suggesting that SOX2 could be suppressing OTX2 (Fig. 5B,C). In terms of cell function, we did not observe significant changes in cell growth (data not shown), but we did observe a significant rescue of self-renewal (Fig. 5D-K) and migration (Fig. 5L-O) for both cell lines following SOX2 overexpression. Given the role of SOX2+ cells in driving SHH MB progression (Ahlfeld et al., 2013; Vanner et al., 2014), we also expected to see effects from SOX2 overexpression alone, independent of an OTX2 phenotypic rescue. No significant changes in primary spheres or migration were detected; however, we observed a small, but significant increase in control Daoy secondary spheres following SOX2 overexpression. Together, these results demonstrate that SOX2 is sufficient to rescue the inhibitory effects of OTX2 on hEN and SHH MB cell self-renewal and migration. The downregulation of OTX2 following SOX2 overexpression suggests that these two transcription factors may participate in a negative feedback loop that regulates cell properties in our model systems.

### OTX2 knockdown decreases cell growth and self-renewal in trans-hENs as well as Group 3 and 4 medulloblastoma cells *in vitro*

**Fig. 6.**
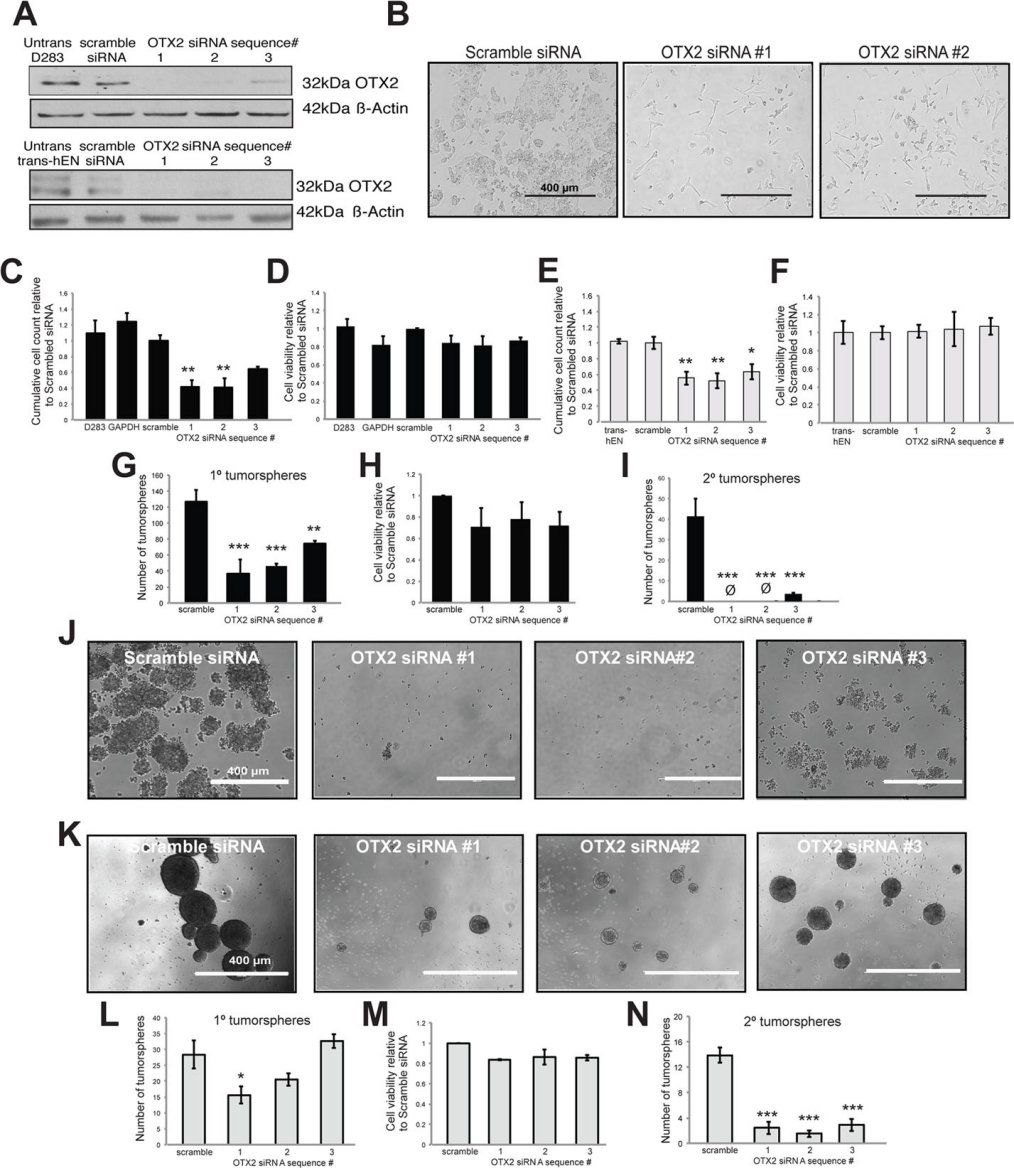
**OTX2 knockdown significantly inhibits cell proliferation and self-renewal in both trans-hENs and D283 MB cells.** (A) Western blot validation of OTX2 knockdown in three independent siRNA sequences relative to scramble siRNA. β-actin serves as a loading control. (B) Representative images of D283 MB cells in adherent culture following 4 days knockdown of OTX2. Scale bars: 400 µm. (C-F) Quantification of total cell number (C,E) and cell viability (D,F) in D283 (C,D) and trans-hENs (E,F) following knockdown of OTX2 over 4 days. (G-N) Self-renewal capacity following OTX2 knockdown in D283 (G-J) and trans-hENs (K-N). In D283, self-renewal capacity was almost completely inhibited following passage to secondary spheres (G-H), and this was evident in representative brightfield images of secondary spheres in (J). (K-M) Significant decreases in neurosphere-forming capacity were also evident in trans-hENs (K,L) following OTX2 knockdown and similar to D283, self-renewal capacity was significantly inhibited (N). For both cell lines, cell viability was not significantly decreased (H,M). Error bars: s.e.m. *P<0.05, **P<0.01, ***P<0.001. For all experiments, *n*=3 biological replicates or independent transfections.

While OTX2 has been shown to induce cell senescence in SHH MB *in vitro* (Bunt et al., 2010), studies involving Group 3 and 4 MB variants have demonstrated an oncogenic role for OTX2 in regulating cell growth (Di et al., 2005; Adamson et al., 2010). To determine whether we could also model this oncogenic role using neural derivatives from hESCs and whether OTX2 also plays a role in other cell properties, we knocked down OTX2 in both D283 Group 4 MB cells and trans-hENs, which resemble Group 3 and 4 MB *in vivo* (Werbowetski-Ogilvie et al., 2012), using small interfering RNAs (siRNAs). We first evaluated the transfection efficiency of D283 and trans-hENs using a Block-iT Red Fluorescent control and demonstrated near 100% transfection of both cell lines after 24 h (supplementary material Fig. S5A-D). Three siRNAs targeting OTX2 were tested, and all three generated a significant knockdown at the protein level relative to scramble siRNA by western blot (Fig. 6A). Following OTX2 knockdown, both D283 MB cells and trans-hENs exhibited a significant decrease in cell growth, and this was not attributed to changes in cell viability (Fig. 6B-F). In addition, knockdown of OTX2 significantly impaired the self-renewal capacity of D283 (Fig. 6G-J) and trans-hENs (Fig. 6K-N) over passage. Knockdown of OTX2 also resulted in a decrease in cell viability, but the results were not significant (Fig. 6H,M). Bromodeoxyuridine staining and analysis supported these findings in sphere culture and demonstrated a decrease in S phase and a concomitant increase in apoptotic cells (supplementary material Fig. S6). We also knocked down OTX2 in D341 (Group 3) MB cells that are exclusively grown in suspension culture or sphere conditions (supplementary material Fig. S5E,F and Fig. S7A). Similar to D283 and trans-hENs, reduction of OTX2 resulted in a significant decline in self-renewal capacity (supplementary material Fig. S7B-E); however, for this cell line, a significant decrease in cell viability was observed (supplementary material Fig. S7D). Evaluation of hESCs genes following OTX2 knockdown revealed significant upregulation of *LIN28A* and *NANOG* only for one siRNA sequence in D283 and trans-hENs (supplementary material Fig. S8A-D). Comparative global gene expression analysis of OTX2 knockdown trans-hENs and trans-hENs confirmed these findings (supplementary material Tables S4 and S5A,B) and revealed a very different list of transcripts from the overexpression results. Transcripts associated with development and differentiation were differentially expressed in OTX2 knockdown trans-hENs (supplementary material Table S5B); however, no differences in hESC genes were observed (supplementary material Table S5A). Collectively, these results demonstrate that trans-hENs effectively recapitulate the findings previously reported for Group 3 and 4 MB lines on cell growth. More importantly, these results demonstrate a novel oncogenic role for OTX2 in maintaining self-renewal of trans-hENs and Group 3 and 4 MB cell lines; however, these effects are independent of pluripotent gene expression.

### OTX2 knockdown decreases *in vivo* tumor growth of trans-hENs and Group 4 medulloblastoma cells

To compare the tumorigenic potential of trans-hENs and D283 MB cells following OTX2 knockdown *in vivo*, we stably knocked down OTX2 using shRNAmir constructs for both cell lines. Following stable selection and validation by western blot (supplementary material Fig. S8E), we confirmed the inhibitory effect of OTX2 knockdown on cumulative cell count and sphere formation seen with the siRNA sequences for both cell lines (supplementary material Fig. S8F-K).

**Fig. 7.**
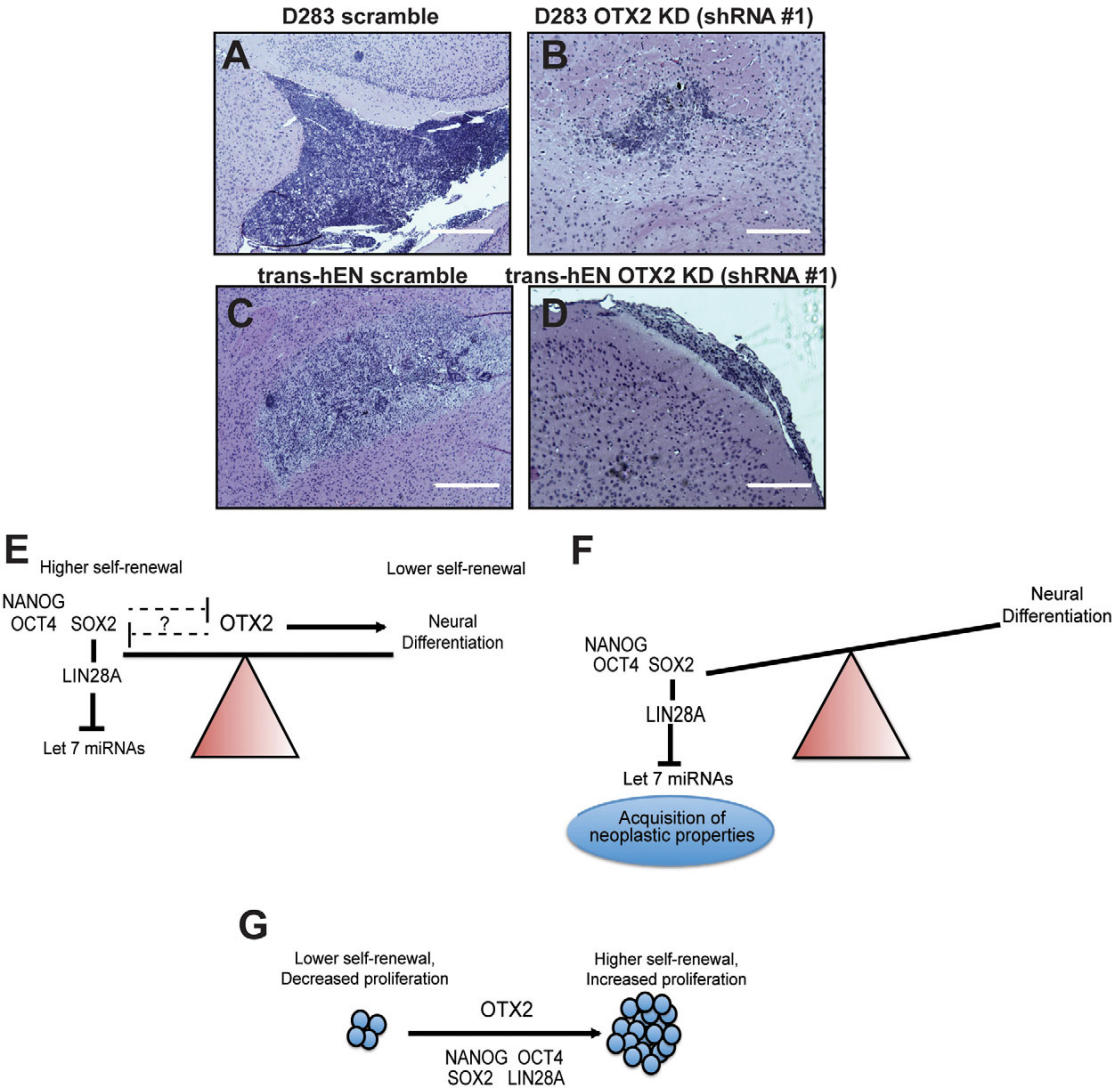
**Knockdown of OTX2 decreases tumor growth *in vivo*, and working models depicting divergent roles of OTX2 and relationship to cell state.** (A-D) Tumorigenic capacity of D283 OTX2 knockdown (KD; A,B) and trans-hEN OTX2 knockdown (C,D) relative to respective scramble control cells in NOD SCID mice. Scale bars: 200 µm. (E,F) The relationship between hESC genes and normal versus aberrant embryonic neural development and how OTX2 expression is predicted to regulate this process in hEN and SHH MB cells. Following OTX2 overexpression, pluripotent genes are downregulated, and our study provides evidence for a relationship between OTX2 and SOX2. (G) The oncogenic role of OTX2 in trans-hENs or Group 3 and 4 MB cells.

Following validations *in vitro*, 5.0×10^5^ (trans-hEN OTX2 knockdown and trans-hEN scramble) and 2.5×10^5^ (D283 OTX2 knockdown and D283 scramble) cells were injected into the right frontal lobe (*n*=5 for each condition and cell line) of NOD SCID mice and examined for tumor formation after 45 days. For both cell lines, knockdown of OTX2 resulted in smaller, but histologically similar tumors compared with respective controls (Fig. 7A-D). For D283, tumors were densely packed, consisting of numerous mitoses in both the dorsal and ventral subarachnoid compartments, and this was accompanied by frequent diffuse invasion into the fourth ventricle. Similar differences in size were seen for trans-hEN cells, with densely packed neural tumors consisting of many neural rosettes and rare mitoses evident in both the striatum and thalamus (Fig. 7A-D). These results support our *in vitro* findings and demonstrate that OTX2 knockdown suppresses trans-hEN and D283 tumor growth *in vivo*.

## DISCUSSION

Our study demonstrates a novel role for OTX2 in self-renewal and migration of human neural precursors and MB cells and identifies a cell-context-dependent link between OTX2 and hESC gene expression. Previous studies in Groups 3 and 4 MB identified many cell cycle and visual perception genes (i.e. *CDKN3*, *HMGB2*, *RP1* and *RBP3*) as direct OTX2 targets (Bunt et al., 2010, 2012), whereas differentiation genes appear to be regulated indirectly by OTX2 (Bunt et al., 2012). Our working model is depicted in Fig. 7E,F, and we propose that OTX2 plays a central role in regulating the earliest stages of neural differentiation to ensure appropriate downregulation of pluripotent genes, such as *SOX2*, and prevent acquisition of tumorigenic properties in SHH MB and hENs. OTX2 and SOX2 may also participate in a negative feedback loop that provides greater control over cellular functions in these cells (Fig. 7E,F). Indeed there are three SOX2 binding sites (TTCAAAG and TACAAAG sequences) on the OTX2 promoter (Ferrari et al., 1992; Okumura-Nakanishi et al., 2005); however, additional studies still need to be conducted to evaluate further potential interactions in our model systems. Recent work by Acampora et al. (2013) supports our model by demonstrating that OTX2 inhibits embryonic stem cell pluripotency and promotes commitment to the epiblast state specifically in mouse cells. Other studies have shown a direct interaction between OTX2 and SOX2 to control ocular development in *Xenopus* (Danno et al., 2008). To our knowledge, however, we are the first to demonstrate an association between OTX2 and SOX2 in regulating self-renewal and migration of human cells. It has recently been shown that SOX2 binds to the *LIN28A* promoter and that a *SOX2-LIN28A-let-7* pathway regulates hESC-derived neural precursor proliferation and differentiation (Cimadamore et al., 2013). In hEN and SHH MB OTX2-overexpressing cells, interaction of OTX2 with SOX2 may prevent subsequent activation of *LIN28A*, resulting in upregulation of *let-7* miRNAs (Fig. 7E,F). Our ChIP studies demonstrated an association between OTX2 and SOX2, but they did not determine whether OTX2 binds directly or indirectly via other proteins to the SOX2 promoter region. Moreover, whether this association is only transcriptional (i.e. OTX2 suppresses hESC gene expression by directly repressing their promoters) or mediated by protein-protein interactions or both in our models is unknown (Fig. 7E). Future studies will evaluate the mechanism(s) by which OTX2 and SOX2 regulate each other and how this interaction between them affects expression of *LIN28A*, downstream target *let-7* miRNAs and other hESC genes to regulate self-renewal and other cellular properties in our models.

While the inhibitory effects of OTX2 in hEN and SHH MB cells are, at least in part, regulated by pluripotent factors, the oncogenic effects of OTX2 on trans-hENs as well as Group 3 and 4 MBs are not mediated by hESC genes (Fig. 7G). Indeed, knockdown of OTX2 resulted in a decrease in cell growth and revealed a novel role for OTX2 in regulating self-renewal of human cells; however, this was accompanied by only modest changes in hESC gene levels that are already elevated in these cells. The context-dependent function of transcription factors is well documented. For example, *OCT4* and *NANOG* are required for maintaining pluripotency; however, their roles change once an hESC acquires tumorigenic features (Ji et al., 2009). Additional studies have shown that both overexpression and knockdown of OCT4 in embryonic stem cells induce differentiation, suggesting that optimal levels are required for maintaining pluripotency (Niwa et al., 2000; Chen et al., 2007). For trans-hENs and Groups 3 and 4 MB, OTX2 may interact with yet unidentified factors to maintain high self-renewal and growth, and this will require further investigation. Additionally, subcellular localization (Baas et al., 2000) and post-translational modifications, including phosphorylation (Kim and Lemke, 2006), may contribute to the differential roles of OTX2 in our model systems and represent important avenues for future study. For example, cell type-specific subcellular localization of Otx2 to the nucleus and/or cytoplasm has been shown to contribute to determination of cell fate during retinal development in the mouse (Baas et al., 2000). Likwise, other homeodomain proteins, such as Vax2, have also been shown to shuttle between the nucleus and cytoplasm during sequential stages of retinal differentiation (Kim and Lemke, 2006).

While our work focused on medulloblastoma as proof of principle, an hESC-based model system can be used to study the role of pluripotency factors and other genes in a diverse range of pediatric neural malignancies. For example, *LIN28A*, a known pluripotent factor and oncogene (Viswanathan and Daley, 2010), has also been found to be a prognostic and diagnostic marker for primitive neuro-ectodermal tumors (Ma et al., 2012) and other rare embryonal brain tumors (Grange et al., 2008). In support of these findings, work in our laboratory has demonstrated that stable overexpression of LIN28A in hENs induces neoplastic features *in vitro*, including enhanced proliferation, neurosphere formation and migration (R.K. and T.E.W.-O., unpublished data). Most recently, Funato et al. (2014) demonstrated that hESC derivatives could not only be used to model diffuse intrinsic pontine gliomas but could also be used for drug screening assays to identify novel pathways that contribute to maintenance of these tumors. The present study reveals a previously unappreciated role for hESCs and their neural derivatives as cellular resources for investigating the molecular mechanisms contributing to MB progression. The use of hENs and trans-hENs circumvents the immediate need for large quantities of expanded cells from heterogeneous MB patient samples, which are very difficult to obtain. They also provide a scalable and reliable *in vitro* surrogate for putative human somatic cancer stem cell populations that can be used in high-throughput screening platforms (Sachlos et al., 2012) prior to moving into validation studies with patient samples. Finally, this model provides a complementary system to genetically engineered mice for studying and validating the function of genes of current clinical interest to the neurooncology community. While our work focused specifically on the functional role of OTX2 both *in vitro* and *in vivo*, neural precursors from hESCs can be used for studying any gene or signaling pathway that regulates cellular processes such as growth, self-renewal and migration. The findings from these studies will have important implications not only for neurodevelopment but also for understanding the fundamental processes contributing to pediatric brain tumor initiation and progression. Using hESCs as both a complement to and a surrogate for existing cell lines and heterogeneous patient samples, the goal is to identify the next generation of targeted therapies that may apply not only to MB, but also to other nervous system cancers, ultimately improving the quality of life for children who survive long term.

## MATERIALS AND METHODS

### Cell culture

Human embryonic stem cells (hESCs; H9) and trans-hESCs (v1; Werbowetski-Ogilvie et al., 2009) were maintained on Matrigel (BD Biosciences, Mississauga, Ontario, Canada) in mTESR™1 with 5X supplement (Stem Cell Technologies, Vancouver, British Columbia, Canada). Confluent cultures were dissociated for 5 min in collagenase IV (Gibco, Invitrogen, Burlington, Ontario, Canada) and passaged 1:4 (trans-hESCs) or 1:2 (hESCs). Neural precursors from normal hESCs (hENs) and their transformed derivatives (trans-hENs) were cultured as previously described (Werbowetski-Ogilvie et al., 2012). Briefly, hESCs and trans-hESCs were dissociated in collagenase IV (Gibco) and re-plated as aggregates onto poly-l-ornithine- and laminin-coated plates (BD Biosciences) in neural media [DMEM/F12 supplemented with 1% N2 (Gibco), 1% B27 (Gibco), 20 ng/ml EGF (BD Biosciences) and 20 ng/ml bFGF (BD Biosciences)]. After 4 days (trans-hESCs) and 6 days (hESCs; passage 0 or start of differentiation), cultures were dissociated into single cells with Accutase (Gibco) and plated back onto laminin-coated plates in neural media for additional passages.

Daoy human MB cells, D283 and D341 cells were purchased from the American Type Culture Collection (ATCC, Rockville, MD, USA). The Daoy MB cell line is derived from a desmoplastic MB (Jacobsen et al., 1985), has been shown to exhibit global activation of SHH-pathway genes (Dahmane et al., 2001; Leung et al., 2004; Wang et al., 2012) and is statistically classified as SHH subgroup based on hierarchical clustering and Principal Component Analysis (PCA) with patient samples (Triscott et al., 2013). D341 (Friedman et al., 1988) and D283 (Friedman et al., 1985; Snuderl et al., 2013) represent Group 3 and Group 4, respectively. Cells were cultured in Eagle's Minimum Essential Medium (ATCC) containing 10% fetal bovine serum (Fisher Scientific, Ottawa, Ontario, Canada). Confluent cultures were dissociated in Accutase (Gibco) and diluted 1:15. For neural precursor differentiation, Daoy, D283 and D341 cells were dissociated for 5 min in Accutase (Gibco) and re-plated in ultralow attachment six-well plates (Corning, Tewksbury, MA, USA) in neural media. Cells were replated every 5 days with media changes every 3 days.

### Self-renewal and migration assays

Briefly, self-renewal was assessed by the ability of cells to be passaged in neurosphere culture at clonal density (5-10 cells/μl) for at least two passages. To assess proliferation, equal numbers of transduced cells (4×10^4^ cells/well) were plated in neural proliferation medium, and the total number of cells was calculated after 4 days in culture. Trypan Blue staining was used to assess cell viability. To evaluate cell migration, aliquots of 2.5×10^4^ cells were prepared as hanging drops in 20 µl as previously described (Werbowetski-Ogilvie et al., 2006). Hanging drops were incubated for up to 4 days to form aggregates and then transferred to collagen type I gels (BD Biosciences) prepared as previously described (Werbowetski-Ogilvie et al., 2006). After collagen gelation at 37°C, embedded aggregates were then overlaid with Eagle's minimum essential medium (ATCC) containing 10% fetal bovine serum (Fisher Scientific). Aggregate measurements were taken at day 0, and migration was measured at 72 h (day 3) using a Zeiss Primo Vert microscope (Carl Zeiss Canada, Toronto, Ontario, Canada) with an ocular micrometer.

### Lentiviral transduction and cell sorting

OTX2 was stably overexpressed in Daoy and/or hENs using purchased Precision LentiORFs (Open Biosystems, Thermo Scientific) consisting of pLOC dual-expression constructs with TurboGFP as a marker to track transduction efficiency and Blasticidin S resistance for stable clone selection. LentiORF RFP-expressing viral particles (from the same backbone pLOC construct) were used as a control. On day 0, 5×10^4^ cells were plated in 24-well format, and incubated overnight. On day 1, medium was removed and virus added using a multiplicity of infection of 0.3. Medium was replaced after overnight incubation. Transduced Daoy cells were treated with blasticidin (Mediatech, Corning) for stable clone selection. For hENs, GFP positives (OTX2+ hENs) and RFP positives (RFP+ control hENs), cells were fluorescent activated cell sorted (FACS) on a MoFloXDP (Beckman Coulter, Inc., Mississauga, Ontario, Canada), re-plated and expanded. OTX2 overexpression was verified by qPCR and western blot.

For SOX2 overexpression, we expanded OTX2+ hEN, OTX2+ Daoy and control cultures to obtain sufficient cell quantities and stably overexpressed SOX2 using ORF cDNA lentiviral particles (GeneCopoeia, Inc., Rockville, MD, USA) consisting of the pReceiver-Lv105 expression vector with a puromycin resistance gene. Negative control lentifect lentiviral particles were used as controls. SOX2 expression was evaluated by western blot. Stably transduced Daoy and hEN cells were subjected to self-renewal, cell migration, proliferation and survival assays using methods previously described (Werbowetski-Ogilvie et al., 2012; Morrison et al., 2013) and available on request.

OTX2 was stably knocked down in trans-hENs and D283 cells using two shRNAmir constructs (Open Biosystems, Inc., Thermo Fisher, Huntsville, AL, USA) consisting of a dual-expression system with TurboGFP as a transduction marker. A nonsilencing (scramble) shRNA sequence was used as a negative control. GFP-positive cells were FACS sorted from transduced cultures 96 h after infection, and OTX2 knockdown was determined by western blot for shRNA sequences #1 and #2. Following stable selection, trans-hEN OTX2 knockdown, D283 OTX2 knockdown and their respective controls were subjected to cumulative cell count, viability and sphere-formation assays.

### Annexin V and cell cycle analysis

To assess cell viability, Annexin V staining was performed as previously described (Ali et al., 2015; Morrison et al., 2013) using a PE Annexin V Apoptosis Detection Kit (BD Biosciences) according to manufacturer's guidelines. Briefly, cells were dissociated, and a single cell suspension was prepared and stained with Annexin V-Allophycocyanin (APC) and 7-amino actinomycin D. For each sample, 1.0×10^5^ cells were stained, incubated for 15 min and then resuspended in 1× binding buffer. Flow cytometry was performed on a Gallios flow cytometer (Beckman Coulter, Inc., Mississauga, Ontario, Canada), and results were analyzed using Kaluza software (Beckman Coulter).

For cell cycle analysis, cells were exposed to 10 µM BrdU for either 1 h (adherent cultures) or 5 h (tumorsphere/neurosphere cultures) prior to harvesting as previously described. Single cell suspensions were prepared (2.0×10^5^ cells/sample), fixed and stained using a BD Pharmingen BrdU Flow Kit (BD Biosciences) according to the manufacturer's guidelines. Cells were counterstained with 7-amino actinomycin D for DNA content, and flow cytometry was performed on a MoFloXDP (Beckman Coulter) cell sorter. Results were analyzed using FlowJo software (Tree Star, Inc., Ashland, OR, USA).

### Intracranial transplants

The University of Manitoba Animal Care Committee approved all procedures and protocols. Dissociated tumorspheres from OTX2+ Daoy and Daoy, as well as trans-hEN OTX2 knockdown, D283 OTX2 knockdown and their respective scramble controls were injected intracranially into NOD SCID mice as previously described (Singh et al., 2004; Werbowetski-Ogilvie et al., 2012). Briefly, 5- to 7-week-old mice were anesthetized with isoflurane and injected in the right frontal lobe with biological replicates consisting of 2.5×10^5^ (Daoy and D283) or 5×10^5^ (trans-hEN) cells. After 45 (D283 and trans-hEN) or 60 days (Daoy; based on signs of tumor formation), animals were anesthetized with isoflurane and perfused with formalin (VWR International, Mississauga, Ontario, Canada) and the brains were extracted, placed in 10% formalin for 2-7 days, embedded in paraffin and then sectioned (5 µm thickness). Sections were de-waxed in xylene, rehydrated through a graded series of alcohol concentrations and stained with hematoxylin and eosin. Slides were mounted and imaged using an EVOS xl core microscope (AMG, Seattle, WA, USA).

### Chromatin immunoprecipitation

Chromatin immunoprecipitation was performed as previously described (Rao et al., 2015). OTX2+ Daoy and Daoy cells (*n*=3 for each) were crosslinked using 1% formaldehyde for 10 min and quenched by 0.125 M glycine for 5 min. Cell pellets were resuspended in 500 μl of sonication buffer [50 mM HEPES, pH 7.9, 140 mM NaCl, 1 mM EDTA, 1% (w/v) Triton X-100, 0.1% (v/v) sodium deoxycholate, 1% (w/v) SDS and protease inhibitors] and sonicated using a bioruptor to obtain fragments ∼400 bp in length. Chromatin was precleared by incubating with protein A-Dynabeads ovalbumin and salmon sperm for 1 h at 4°C. Antibodies (anti-trimethyl H3K4, anti-trimethyl H3K27 and OTX2) were coupled to Dynabeads with protein A (for rabbit antibodies) or with protein G (for mouse antibodies) for 2 h at room temperature in immunopreciptation buffer. Precleared chromatin was added to antibody-bound beads and incubated overnight at 4°C. Unbound chromatin was washed with wash buffer A [50 mM HEPES, pH 7.9, 500 mM NaCl, 1 mM EDTA, 1% (w/v) Triton X-100 and 0.1% (v/v) sodium deoxycholate], wash buffer B (20 mM Tris pH 8.0, 1 mM EDTA, 250 mM LiCl and 0.5% NP40) and TE. The bound fraction was eluted in 400 μl of elution buffer and reverse crosslinked with 5 M NaCl (0.2 M final) and 4 μl of RNAse A (1 mg/ml) overnight at 55°C. Samples were treated with proteinase K (20 μg/μl) for 2 h at 42°C. DNA was extracted by the phenol:chloroform method and then suspended in 50 μl of elution buffer. Immunoprecipitated DNA was subjected to qPCR using specific primer sets based on promoter or 5′ sequences (supplementary material Table S6) for each gene interrogated using the Ensembl Genome Browser (*SOX2*: ENST00000325404, NM_003106; *NANOG*: ENST00000229307, NM_024865; *OCT4*: ENST00000513407, NM_001285986; and *LIN28A*: ENST00000326279, NM_024674) and the percentage of material immunoprecipitated was analyzed using the following formulae: (1) DNA Input=2^40−Input *Ct*^×1000; (2) Percentage of material immunoprecipitated or percentage recovery from immunoprecipitation=2^40−IP *Ct*^/DNA Input×100.

### Luciferase assay

OTX2+ Daoy, OTX2+ hENs and their respective control cells were subjected to luciferase assays using the Secrete-Pair Gaussia Luciferase Assay kit (SPGA-G100; Gene Copoeia) according to the manufacturer's guidelines. Briefly, OTX2+ Daoy and OTX2+ hENs along with RFP control cells (5.0×10^4^) were transfected with SOX2 promoter reporter clone (HPRM15202-PG02). Cells were also transfected with the positive GAPDH-PG02 and negative-PG02 control clones. Culture media was collected 72 h after transfection, and luciferase activity was measured in 10 μl of media on an LMAX luminometer (Molecular Devices) with 100 μl of luciferase assay substrate (Gene Copoeia). Negative control values were subtracted from all data points to eliminate background signal. Data were normalized to the control for each cell line and presented as the fold change in luciferase activity.

### Western blot

Total protein was isolated from hENs, trans-hENs, Daoy, D283 and D341 MB cell lines using the All-in-One Purification Kit (Norgen Biotek, Thorold, Ontario, Canada) according to the manufacturer's instructions. Protein samples consisting of 10 μg were run on a 12% Tris-glycine gel and subsequently transferred onto a 0.45 μm nitrocellulose membrane (Bio-Rad Laboratories, Mississauga, Ontario, Canada). OTX2 and SOX2 blots were treated with SuperSignal Western Blot Enhancer (Thermo Scientific) and then incubated in 2% skim milk overnight. Blots were incubated in primary rabbit anti-OTX2 (1:500 dilution; Abcam, ab21990) or rabbit anti-SOX2 antibody (1:500 dilution; Cell Signaling Technologies, #3579) for 2 h at room temperature. β-actin (Sigma) was used as a loading control. Blots were treated with secondary goat-anti-rabbit antibody (1:5000 dilution; Bio-Rad Laboratories) for 1 h at room temperature. Super Signal West Pico Chemiluminescent substrate (Thermo Scientific) was used for detection.

### Small interfering RNA

OTX2 levels were knocked down in trans-hEN, D283 and D341 cells using Silencer select siRNAs (Life Technologies, Burlington, Ontario, Canada). A nonsilencing (scrambled) siRNA and GAPDH siRNA were used as a negative and positive control, respectively. Three independent siRNAs targeting OTX2 (s9931, s9932 and s9933) were evaluated, and all sequences were used at a final concentration of 20 nM (trans-hENs) and 30 nM (D283). Knockdown of OTX2 was assessed by western blot.

### TaqMan expression assays (miRNA or MicroRNA)

RNA was extracted using the miRVana isolation kit following the manufacturer's instructions (Life Technologies). qRT-PCR validation of selected miRNA was performed using TaqMan primer/probes for let-7i, let-7d and let-7c, the MicroRNA Reverse Transcription Kit and TaqMan Universal Master Mix II, with uracil-N-glycosylase and performed on a Mx3000P (Stratagene) qPCR system. Results were normalized to the U6 small nuclear RNA endogenous control using the Δ*Ct* method.

### Gene expression profiling

Extracted RNA from OTX2+ hEN and hEN neurospheres (*n*=3 biological replicates) as well as OTX2 knockdown trans-hEN and trans-hEN neurospheres (*n*=2 biological replicates with sequence #3) was subjected to GeneChip 3′ oligonucleotide microarray hybridization and processing performed by the London Regional Genomics Centre (Robarts Research Institute, London, Ontario, Canada) according to manufacturer's protocols (Affymetrix). Ten micrograms of cRNA was labelled and hybridized to Affymetrix Human Gene 2.0 ST chips. Expression signals were scanned on an Affymetrix GeneChip Scanner, and data extraction was performed using Affymetrix AGCC software. Data normalization and analysis were performed using Partek software (Partek Inc., St Louis, MO, USA). Hierarchical clustering using Pearson correlation coefficients was performed on the normalized data. Differentially expressed genes were analyzed using Ingenuity Pathway Analysis (IPA; Redwood City, CA, USA). Transcripts differentially expressed at least 1.5-fold (up- or downregulated) and with a value of *P*<0.05 were considered significant.

### qRT-PCR

Cell populations were subjected to qPCR analysis of *OTX2*, *LIN8A*, *NANOG*, *SOX2*, *OCT4*, *MMP1*, *TIMP4*, *CNTN1*, *UNC5C*, *FOS*, *FOSB* and *IGF1* transcript levels. Total RNA was extracted using the Norgen all-in-one kit (Norgen Biotek) according to the manufacturer's guidelines. First-strand cDNA was synthesized using the Superscript III First Strand Synthesis System (Invitrogen). The following PCR conditions were used: 50°C for 2 min, 95°C for 2 min, and 40 cycles of 95°C for 15 s, 60°C for 30 s. Quantitative PCR was conducted using GoTaq qPCR Master Mix (Fisher Scientific) and performed on a Mx3000P (Stratagene) qPCR system. All values were normalized to *GAPDH*. Specific primer sequences for each gene are listed in supplementary material Table S6.

### Statistical analysis

All tests were performed using Prism 5 software (GraphPad Software, La Jolla, CA, USA). Descriptive statistics were used to determine significant differences, including mean and standard error of the mean along with one-way ANOVAs, independent-sample two-tailed *t*-tests and Tukey's test for multiple comparisons. Values of *P*<0.05 were considered significant.
